# Genome-wide Association Studies of the Pathogenic Sphingosine-1-Phosphate Gene in Ulcerative Colitis

**DOI:** 10.2174/0118715303354936250523030225

**Published:** 2025-05-29

**Authors:** Hongyu Chen, Jingyu Shang, Song Zhao, Luzhou Xu, Hong Shen

**Affiliations:** 1 Department of Gastroenterology, Affiliated Hospital of Nanjing University of Chinese Medicine (JiangSu Province Hospital of Chinese Medicine), Nanjing, China

**Keywords:** Ulcerative colitis, sphingosine-1-phosphate, *SPHK2*, *SPNS2*, immune cell infiltration, neutrophils

## Abstract

**Background:**

Ulcerative colitis (UC) is a chronic inflammatory bowel disease that can lead to malignancies over time. Sphingosine-1-phosphate (S1P) receptor signaling affects lymphocyte trafficking and vascular integrity, influencing intestinal inflammation. This study aimed to identify S1P-related key genes in UC.

**Methods:**

Differentially expressed genes (DEGs) between the UC and control groups were analyzed in the GSE87473 (training) dataset. Genes overlapping between the DEGs and S1P-related genes were considered candidate genes. These genes were incorporated into machine learning algorithms and subjected to expression analysis to identify key genes. Gene functions were determined through a gene–gene interaction network, enrichment analysis, and immune cell infiltration analysis. In addition, transcription factor–mRNA and mRNA–miRNA–lncRNA networks were constructed. Finally, reverse transcription–quantitative polymerase chain reaction (RT-qPCR) was performed to evaluate the expression of key candidate genes in UC and control tissues.

**Results:**

This study identified two key genes (SPHK2 and SPNS2) associated with UC. Notably, *SPHK2* expression was lower and *SPNS2* expression was higher in the UC group in both training and validation datasets and in clinical UC tissues (RT-qPCR). The area under the curve values of *SPHK2* and *SPNS2* exceeded 0.7 in both datasets, indicating that the genes had good diagnostic efficacy for UC. Consistently, the nomogram showed that the two genes had promising diagnostic value in UC. *SPHK2* and *SPNS2* were found to be localized to the plasma membrane. The correlations of the two genes with different immune cells showed significantly opposite trends. In particular, *SPHK2* had the strongest positive correlation with M2 macrophages (r = 0.6) and the strongest negative correlation with neutrophils. Moreover, mRNA–miRNA–lncRNA and transcription factor–mRNA networks of the key genes were constructed.

**Conclusion:**

This study suggests that *SPHK2* and *SPNS2* are key genes associated with UC, highlighting their potential as effective diagnostic biomarkers.

## 1 INTRODUCTION

Ulcerative colitis (UC) is a chronic, relapsing inflammatory bowel disease (IBD) characterized by mucosal inflammation, typically initiating in the distal colon and progressing proximally [1]. Common clinical manifestations include persistent or recurrent diarrhea, mucopurulent and bloody stools, as well as systemic symptoms of varying severity [2]. In 2023, the global incidence of UC reached approximately 5 million cases, with an increasing prevalence [3]. Colonoscopy remains the gold standard for UC monitoring and assessment [4]. UC exhibits a bimodal age distribution, with peak incidences observed at ages 20–30 and 50–80 years [1]. Chronic UC can lead to intestinal complications, and prolonged disease duration increases the risk of inflammation-associated malignancies [5]. UC represents a significant global health burden, with a higher incidence in developed countries and an increasing prevalence in developing nations [6]. Risk factors for UC include genetic predisposition, environmental influences, dysbiosis, and immune dysregulation [7]. The primary therapeutic goals for UC management are symptom control, induction and maintenance of remission, mucosal healing, and prevention of disease recurrence. Despite ongoing efforts to expand therapeutic options, the management of UC remains challenging, and its pathogenesis remains incompletely understood. Identifying novel biomarkers could improve diagnostic accuracy and therapeutic strategies [8].

Sphingosine-1-phosphate (S1P) is a bioactive sphingolipid metabolite composed of a hydrophobic fatty acid chain and a phosphate group, which plays a pivotal role in various cellular processes, including proliferation, migration, differentiation, and inhibition of apoptosis [9]. The sphingosine kinases responsible for S1P synthesis play dual roles in intracellular lipoprotein metabolism [10] and extracellular signaling pathways [11]. S1P signaling is mediated by five G protein-coupled receptors: S1PR1, S1PR2, S1PR3, S1PR4, and S1PR5 [12]. Sphingosine kinase 1 (SPHK1) and sphingosine kinase 2 (SPHK2) catalyze the phosphorylation of sphingosine to produce S1P. SPHK1 is primarily localized in the cytoplasm and plasma membrane, where it rapidly generates S1P upon stimulation [13], whereas SPHK2, located in the endoplasmic reticulum [14], produces S1P in a more sustained and regulated manner. Following its synthesis, S1P is exported from the cell *via* transporters such as SPNS1 and ABCC1 [15, 16]. Extracellular S1P is metabolized by enzymes such as S1P lyase, which degrades it into hexadecenoaldehyde and ethanolamine phosphate [17, 18], and by S1P phosphatase, which converts it back into sphingosine [19], thus regulating its signaling and maintaining cellular homeostasis. Although the reactions catalyzed by these enzymes are similar, their structural characteristics and subcellular localization differ. In comparison to S1PR2, SPHK1 is more highly expressed at the plasma membrane [20], where it regulates extracellular S1P signaling and processes such as migration, angiogenesis, and tissue repair [21, 22]. In contrast, SPHK2 is predominantly localized intracellularly [23-25] and is implicated in stress responses and apoptosis regulation [22, 26]. These structural and localization differences enable SPHK1 and SPHK2 to mediate distinct and context-dependent functions, which are of critical importance in both physiological and pathological conditions. Due to its broad physiological impact, S1P is involved in the pathogenesis of various diseases, including cardiovascular disorders, multiple sclerosis, kidney disease, and osteoporosis [27-30]. The S1P receptor signaling system plays a crucial role in lymphocyte trafficking [31], vascular integrity [22], and the regulation of intestinal inflammation [32], although the precise molecular mechanisms remain to be fully elucidated.

Given the significant role of S1P in UC pathogenesis, the roles of S1P-related genes in regulating metabolic functions [33] and immune responses [34] warrant investigation to identify potential diagnostic biomarkers and therapeutic targets for UC. In this study, we integrated transcriptomic data from UC patients available in public databases to identify S1P-related genes associated with UC. Bioinformatic tools were employed to construct diagnostic models and analyze the potential molecular mechanisms of these genes. The results of this study may provide valuable insights for the development of novel diagnostic and therapeutic strategies for UC in clinical settings.

## 2 MATERIALS AND METHODS

### 2.1 Data Sources

UC-related datasets were downloaded from the Gene Expression Omnibus database (https://www.ncbi.nlm.nih.gov/geo/). The GSE87473 dataset (training set), based on the GPL13158 sequencing platform, included 21 normal and 106 diseased mucosal tissue samples from patients with UC. The GSE48958 dataset (validation set), based on the GPL6244 sequencing platform, comprised 8 normal and 7 diseased mucosal tissue samples from patients with UC. In addition, “GOBP_SPHINGOSINE_1_PHOSPHATE_RECEPTOR_SIGNALING_PATHWAY” was searched on the Molecular Signatures Database (MSigDB, https://www.gsea-msigdb.org/), and data on 15 S1P-related genes (SRGs) were obtained (Table **S1**). The information on the databases used in this study can be found in Table **S2**.

### 2.2 Differential Expression Analysis

The limma (v 3.54.0) package [35] was used to identify differentially expressed genes (DEGs) between the UC and control groups in the training set based on the following screening criteria: |log_2_fold-change (FC)| > 0.5 and *p* < 0.05. Subsequently, the clusterProfiler (v 4.2.2) package was used to perform Gene Ontology (GO) and Kyoto Encyclopedia of Genes and Genomes (KEGG) analyses on the DEGs, with a *p*-value of <0.05 indicating statistical significance [36].

### 2.3 Selection and Analysis of Candidate Genes

DEGs were intersected with SRGs, and the overlapping genes were identified as candidates for further analysis. The STRING database (https://cn.string-db.org/) was used to construct a protein–protein interaction (PPI) network (interaction degree = 0.15) of the candidate genes to analyze the relationship between the genes at the protein level.

### 2.4 Machine Learning and Expression Analyses of Key Genes

The glmnet (v 4.1-4) package was used to perform least absolute shrinkage and selection operator (LASSO) analysis on candidate gene expression in the training set, resulting in the generation of a feature gene set (feature genes1) [37]. Simultaneously, the e1071 (v 1.7-13) package was used to implement support vector machine-recursive feature elimination (SVM-RFE) analysis [38] to generate another feature gene set (feature genes2). After the two gene sets were intersected, the overlapping genes were identified as candidate key genes. The expression profiles of the candidate key genes were compared between the UC and control groups in the training and validation sets. The genes significantly expressed in the UC and control groups had similar expression trends and were considered key genes. Receiver operating characteristic (ROC) curves were generated to assess the diagnostic efficacy of each key gene in the two datasets. The area under the curve (AUC) values of all genes exceeded 0.7, indicating that the genes had good diagnostic value.

### 2.5 Construction and Validation of a Diagnostic Nomogram

To examine the relationship between the key genes and UC, a diagnostic nomogram was constructed based on the multivariate logistic regression model using the rms (v 6.5.0) package. The expression of each key gene was analyzed to obtain a corresponding score, and the incidence of UC was evaluated based on the total score obtained. In addition, calibration and ROC curves were plotted to validate the predictive accuracy of the nomogram.

### 2.6 Functional Analysis of Key Genes

Data on the subcellular localization of key genes were obtained from the GeneCards database. Genes with a confidence value of ≥3 were assembled into a subcellular localization network, and the chromosomal locations of the key genes were identified. A gene–gene interaction network of the key genes and key gene-related pathways was analyzed using the GeneMINIA database. The clusterProfiler and org.Hs.eg.db packages were used to perform single-sample gene set enrichment analysis (GSEA) on the key genes. In particular, all genes were ranked based on the correlation between the key genes and the expression of all genes in the training set, with the screening thresholds set at |NES| of >1, NOM *p*-value of <0.05, and *q*-value of <0.25.

### 2.7 Immune Infiltration Analysis

Samples from the training set were analyzed for immune infiltration. The distribution scores of 22 types of immune cells were evaluated in each sample using the CIBERSORT algorithm. The rank-sum test was used to compare immune cell infiltration between the UC and control groups, with a *p*-value of <0.05 indicating statistically significant differences. Furthermore, Spearman correlation analysis was performed to assess the relationship between the key genes and the immune cells with significantly different infiltration levels.

### 2.8 Upstream Regulatory Networks of Key Genes

miRNAs and lncRNAs associated with the key genes were predicted using the starBase database, which integrates several databases. The miRmap database was used to ensure that miRNAs were predicted for all key genes. Subsequently, miRNA-associated lncRNAs were predicted using a clipExpNum value of ≥20 as the criterion. The ChEA3 database was used to identify the transcription factors (TFs) of the key genes, and the Cytoscape software was used to construct a TF–mRNA network.

### 2.9 Expression Analysis of Key Genes

To validate the expression of the key genes, reverse transcription–quantitative polymerase chain reaction (RT-qPCR) was performed on 5 UC and 5 control tissue samples. The conditions for qPCR were as follows: 40 cycles at 95ºC for 1 min, 95ºC for 20 s, 55ºC for 20 s, and 72ºC for 30 s. The primer sequences used for qPCR are listed in Table **S1**. The relative RNA expression of the key genes was calculated using the 2^-∆∆CT^ method, with GAPDH serving as the internal reference gene.

## 3 RESULTS

### 3.1 Six Candidate Genes were Identified upon Overlapping 3,514 DEGs with 15 SRGs

**Fig. (1):**
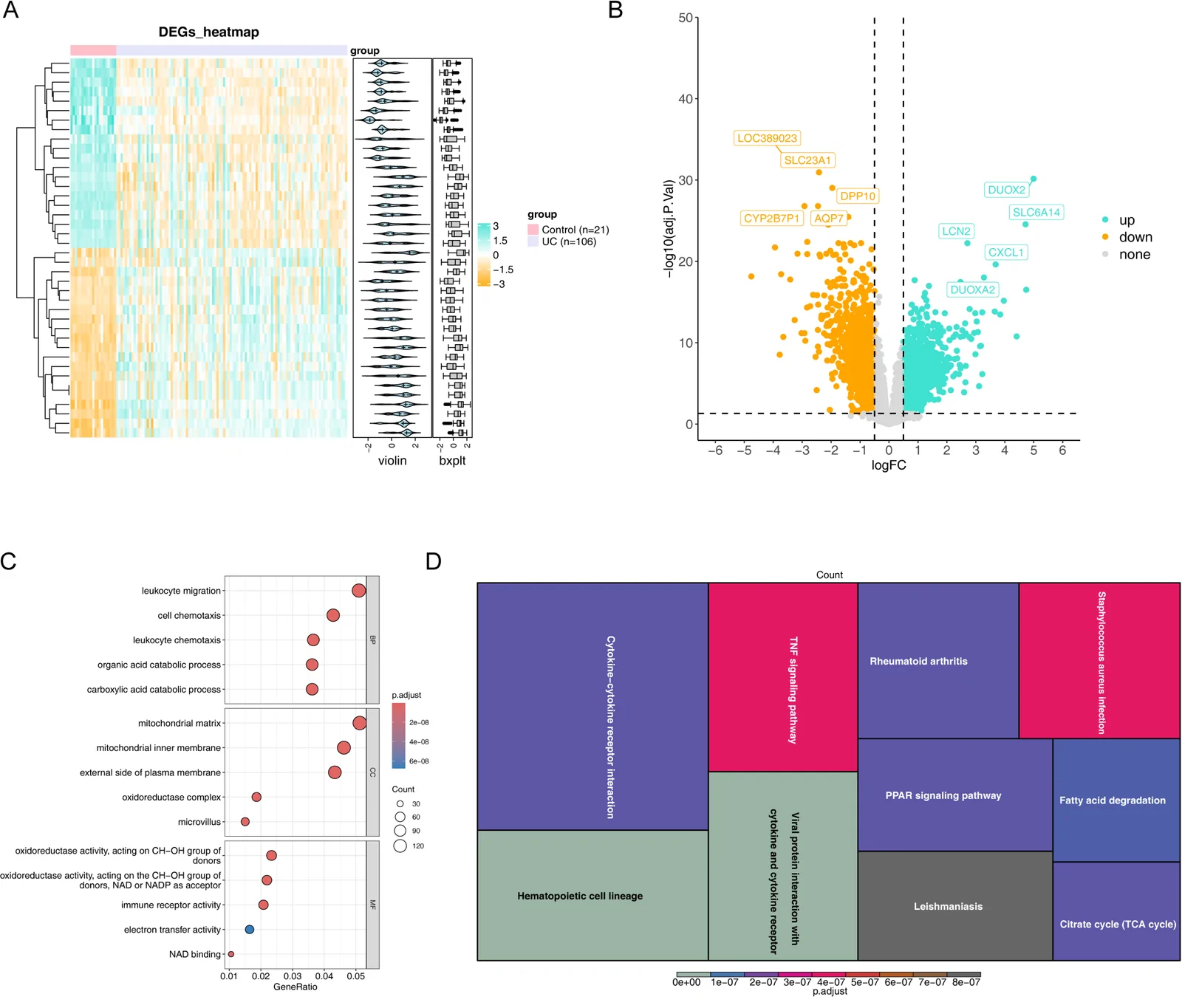
Differential expression analysis based on the sequencing data of mucosal biopsy samples from patients with UC in the training set. (**A**). Volcano plot of differentially expressed genes. (**B**). Heatmap of differentially expressed genes. (**C**). Bubble plot of Gene Ontology (GO) terms associated with differentially expressed genes. (**D**). Module diagram of Kyoto Encyclopedia of Genes and Genomes (KEGG) pathways associated with differentially expressed genes.

**Fig. (2):**
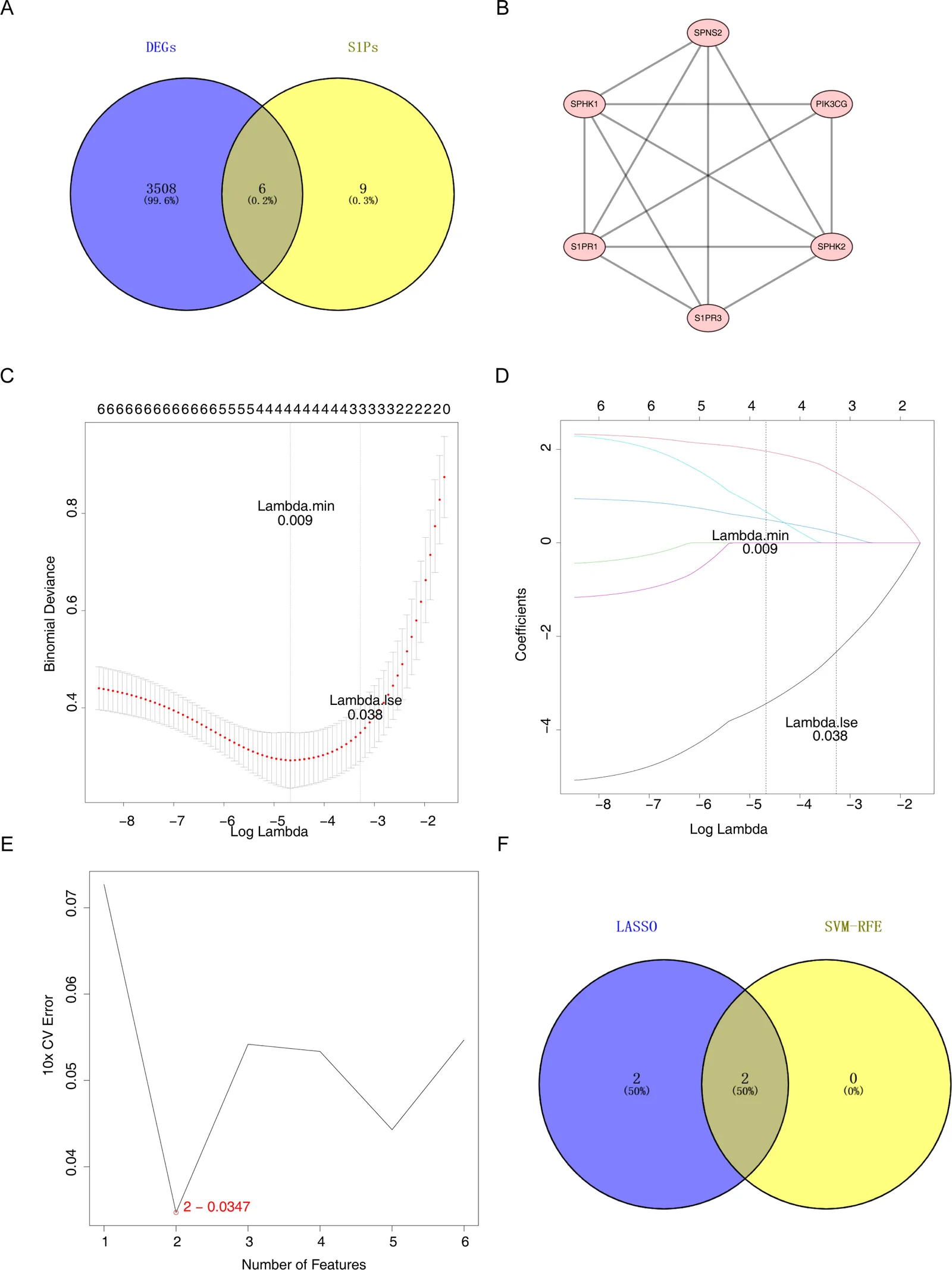
Screening of key genes associated with UC. (**A**). Venn diagram of candidate genes. (**B**). PPI network of candidate genes. (**C**). Seven-fold cross-validation for tuning parameter selection in the LASSO model, with a lambda.min value of 0.009. (**D**). LASSO coefficient profiles. (**E**). Ten-fold cross-validation for tuning parameter selection in the SVM-RFE model. (**F**). Venn diagram of key genes.

A total of 3,514 DEGs, with 1,407 upregulated and 2,107 downregulated genes, were identified between the UC and control groups in the training set (Figs. **1A**, **B**). GO analysis showed that the DEGs were enriched in leukocyte migration, oxidoreductase complex, and immune receptor activity. In addition, KEGG analysis showed that the DEGs were enriched in pathways associated with leishmaniasis, tumor necrosis factor (TNF) signaling, and citrate cycle (Figs. **1C**, **D**). The intersection of the 3,514 DEGs with 15 SRGs revealed six candidate genes (Fig. **2A**). The PPI network of the six candidate genes revealed 13 inter-relationships between the genes (Fig. **2B**).

### 3.2 *SPHK2* and *SPNS2* had Excellent Diagnostic Efficacy for UC

**Fig. (3):**
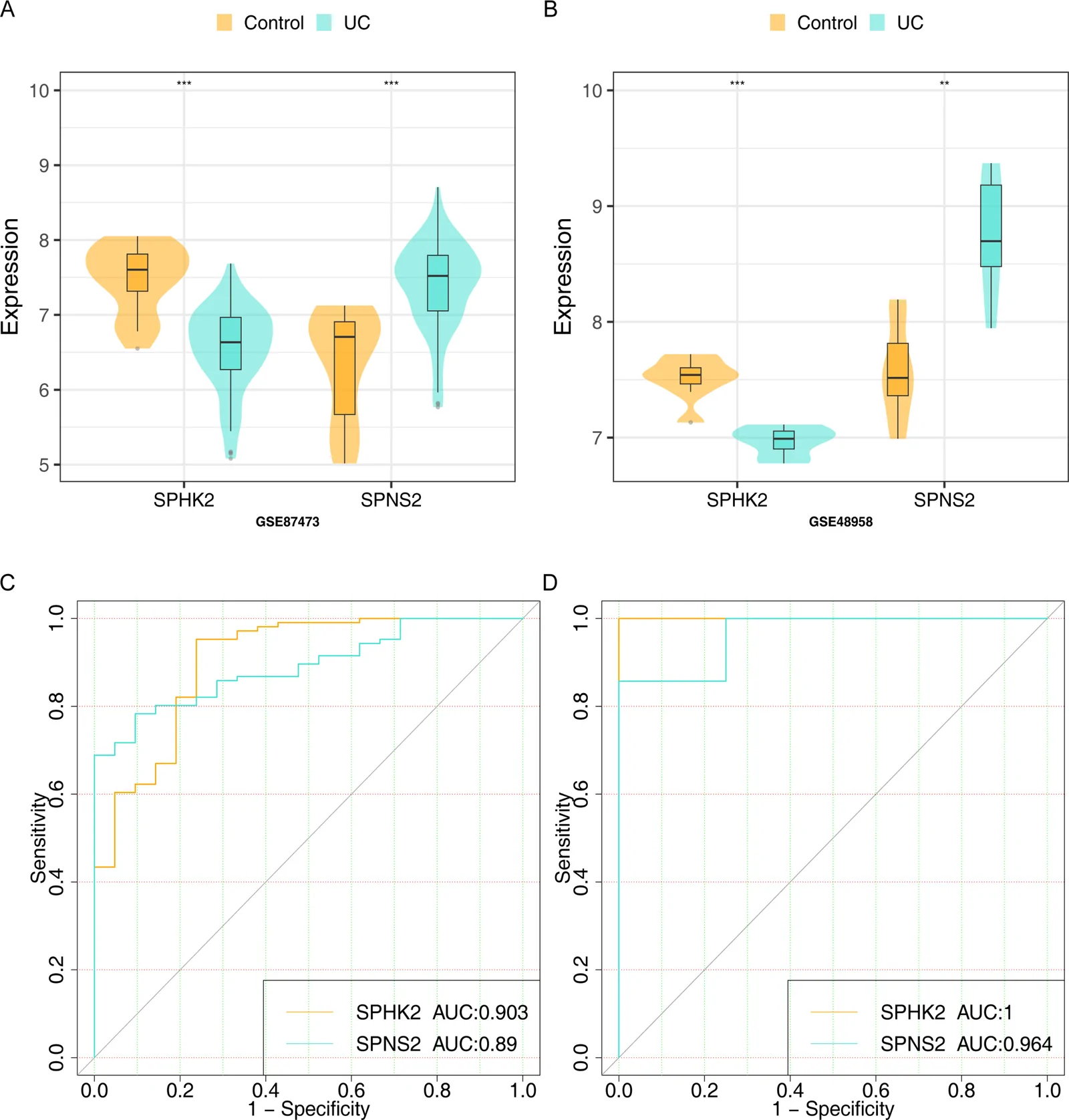
Validation of key genes associated with UC. (**A, B**). Box plots of the expression of the key genes in the GSE87473 and GSE48958 datasets. The asterisks indicate the levels of statistical significance: *, *p* < 0.05; **, *p* < 0.01; ***, *p* < 0.001. (**C, D**). Box plots of the expression of the key genes evaluated *via* RT-qPCR.

LASSO regression analysis revealed four featured genes (feature genes1) (Lambda.min = 0.009) (Figs. **2C**, **D**), whereas SVM-RFE analysis revealed two feature genes (featured genes2), with an error rate of 0.0347 (Fig. **2E**). The two feature gene sets were intersected, resulting in the identification of two candidate key genes, namely, SPHK2 and SPNS2 (Fig. **2F**). The two candidate key genes showed differential expression between the UC and control groups in the training and validation datasets and had consistent expression patterns in both datasets. Therefore, the two genes were considered key genes for subsequent analysis and validation (Figs. **3A**, **B**). RT-qPCR showed that the UC group had low *SPHK2* expression and high *SPNS2* expression in both training and validation sets (Figs. **3C**, **D**). The AUC values of *SPHK2* and *SPNS2* were 0.903 and 0.89 in the training set and 1 and 0.964 in the validation set, respectively. These findings indicated that both key genes had excellent diagnostic efficacy for UC.

### 3.3 Nomogram Exhibited Excellent Diagnostic Value in UC

**Fig. (4):**
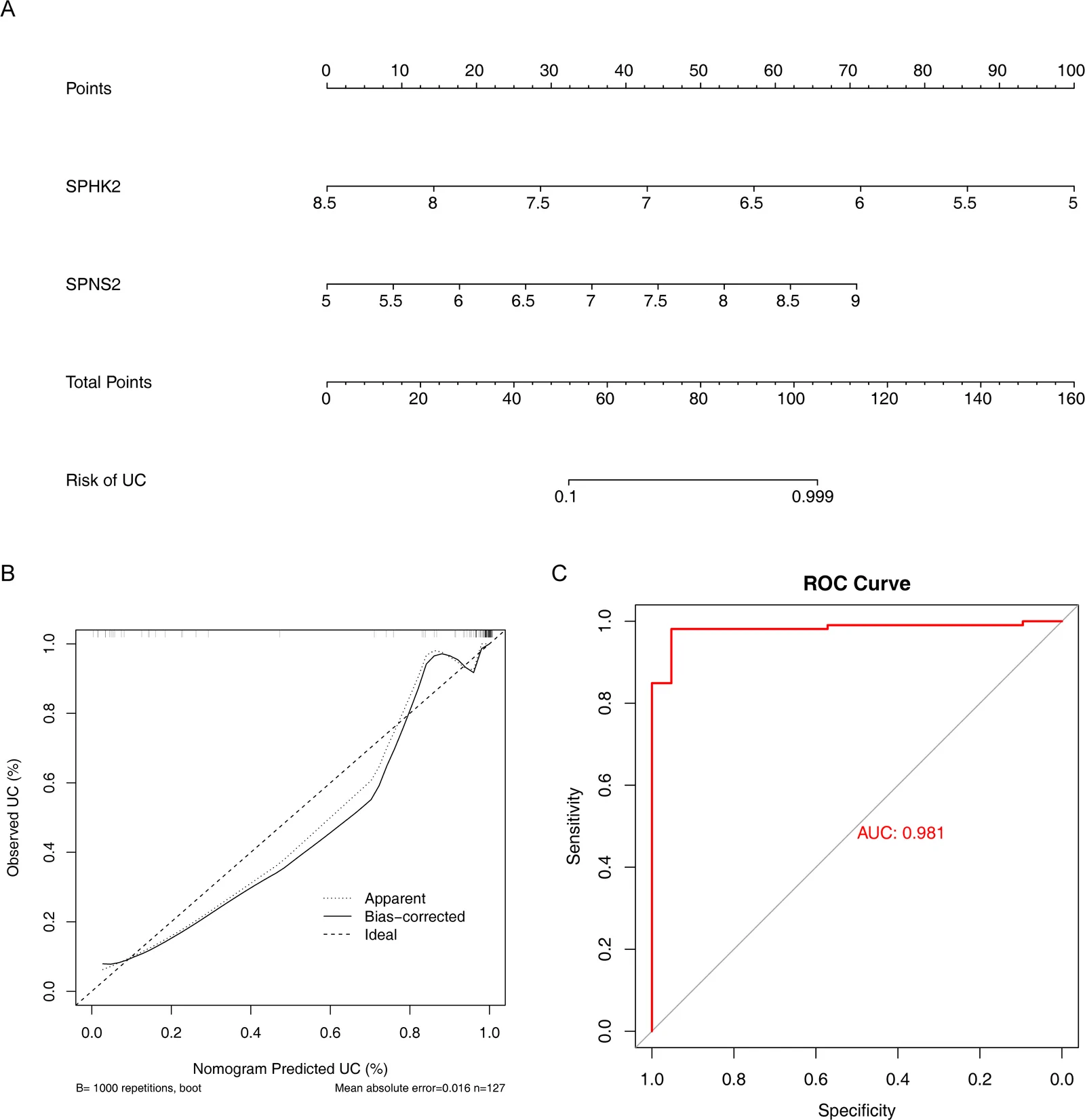
Construction of a nomogram model based on key genes to predict the risk of UC. (**A**). Construction of a nomogram model based on the expression levels of the key genes in the UC and control groups. (**B**). Construction of a calibration curve for assessing the predictive performance of the nomogram model. (**C**). ROC curve for assessing the predictive performance of the nomogram model.

A nomogram based on *SPHK2* and *SPNS2* expression levels was constructed to predict the risk of UC (Fig. **4A**). As shown in the calibration curve in Fig. (**4B**), the bias-corrected curve was close to the ideal curve. Moreover, the AUC value of the ROC curve was 0.981, which indicated that the nomogram had excellent predictive performance (Fig. **4C**).

### 3.4 *SPHK2* and *SPNS2* were Localized to the Plasma Membrane

**Fig. (5):**
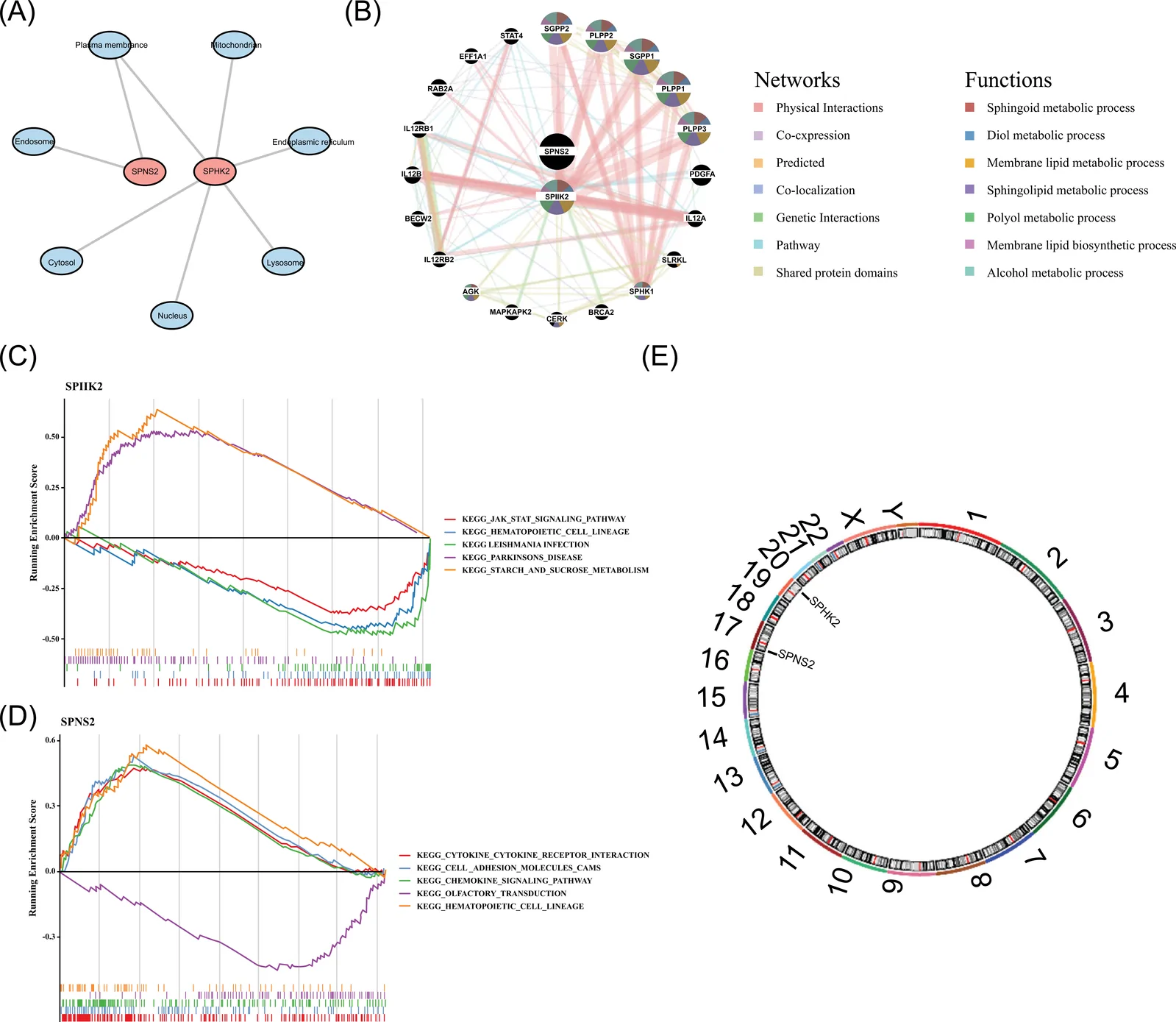
Functional analysis of the two key genes. (**A**). Subcellular localization network of the key genes. (**B**). GeneMANIA network. (**C, D**). KEGG enrichment analysis and GSEA of each key gene based on the correlation of SPHK2 and SPNS2 with the expression levels of all genes in the dataset. (**E**). Circos plot of the chromosomal localization of the key genes.

As shown in Fig. (**5A**), *SPHK2* and *SPNS2* were found localized to the plasma membrane. The gene–gene interaction network showed that *SPHK2* and *SPNS2* interacted with 20 peripheral genes (Fig. **5B**). KEGG enrichment analysis showed that *SPHK2* was enriched in 71 pathways, including those related to JAK–STAT signaling, hematopoietic cell lineage, and starch and sucrose metabolism (Fig. **5C**), whereas *SPNS2* was enriched in 62 pathways, including the chemokine signaling pathway (Fig. **5D**). Chromosomal localization analysis suggested that *SPHK2* was located on chromosome 19 and *SPNS2* was located on chromosome 17 (Fig. **5E**).

### 3.5 *SPHK2* and *SPNS2* had Contrasting Correlations with Immune Cells

**Fig. (6):**
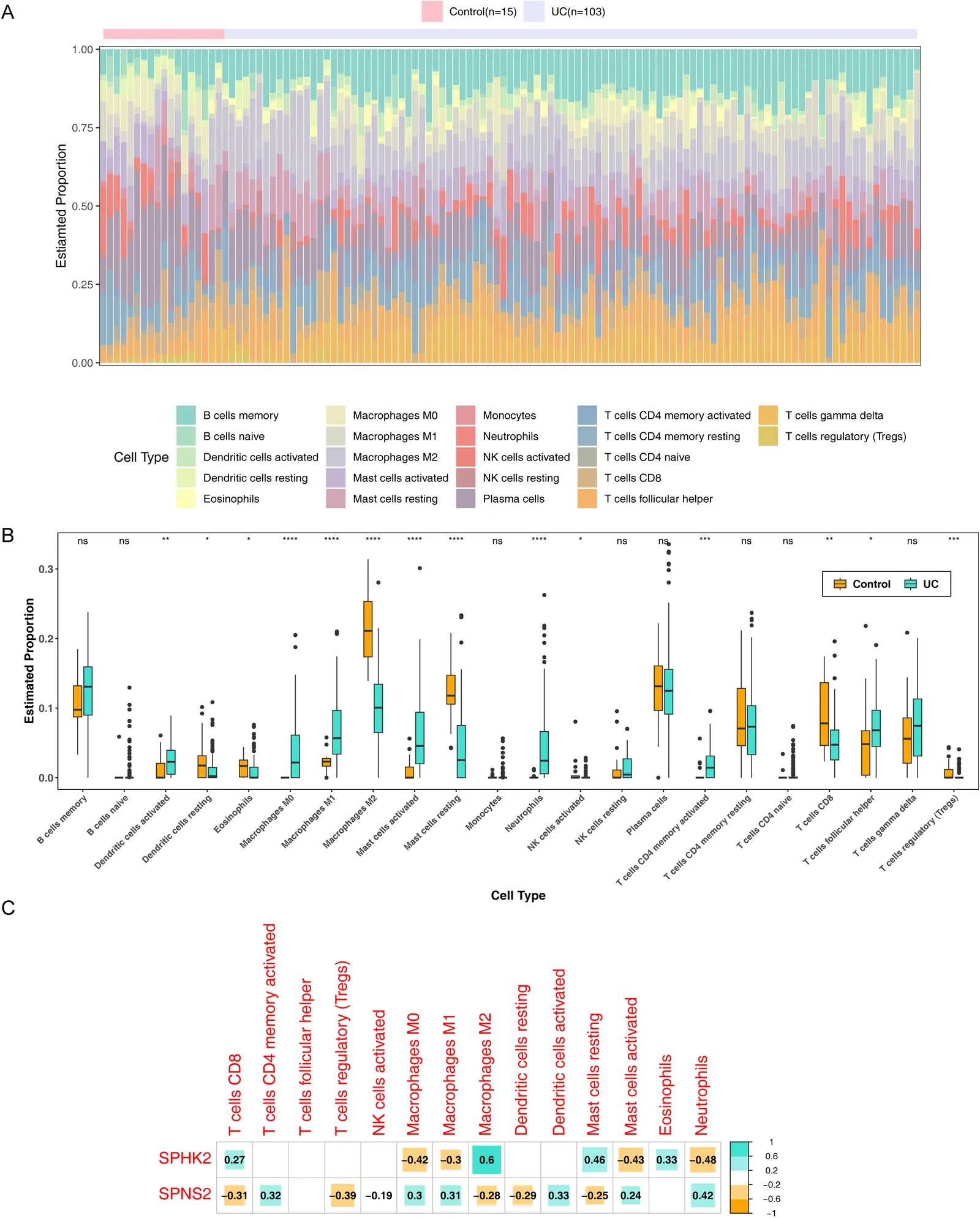
Immune cell infiltration analysis. (**A**). Stacked bar plot of the proportions of immune cells. (**B**). Boxplot of the proportions of immune cells. (**C**). Heatmap of Spearman correlation analysis of key genes and immune infiltration scores. * , *P* < 0.05; **P < 0.01, ***P < 0.001 ****, *P* < 0.0001.

Fig. (**6A**) presents the distribution of scores of 22 immune cell types in the UC and control groups. The proportions of 14 types of immune cells significantly differed between the UC and control groups (*p* < 0.05). These cells included M0, M1, and M2 macrophages (Fig. **6B**). The correlations between *SPHK2* and differential immune cells were remarkably contradictory to those between *SPNS2* and the immune cells. In particular, *SPHK2* had the strongest positive correlation with M2 macrophages (r = 0.6) and the strongest negative correlation with neutrophils (r = -0.48). On the contrary, *SPNS2* had the strongest positive correlation with neutrophils (r = 0.42) and the strongest negative correlation with regulatory T cells (r = -0.39) (Fig. **6C**).

### 3.6 mRNA–miRNA–lncRNA and TF–mRNA Networks

**Fig. (7):**
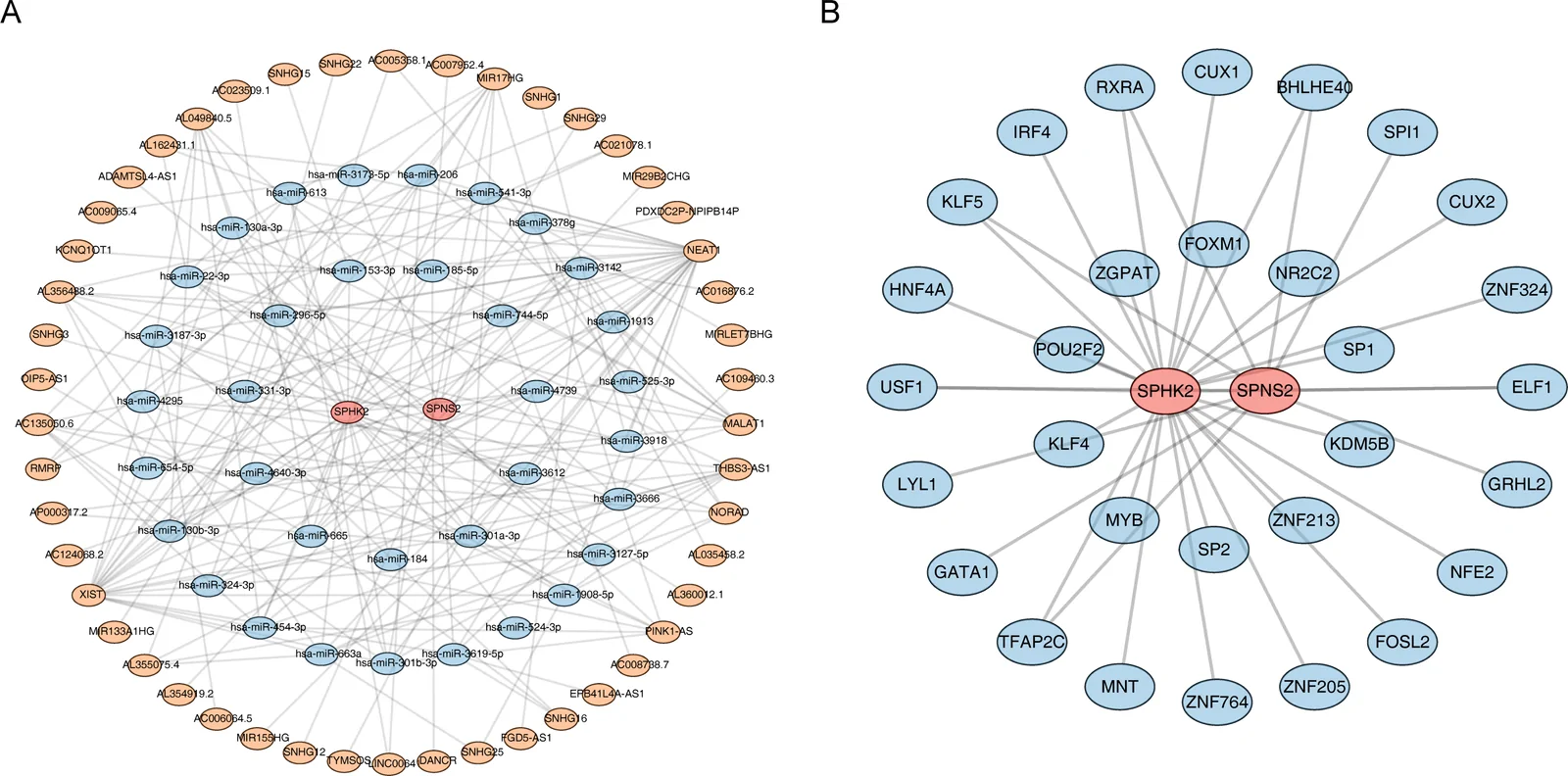
Transcriptional regulatory network of the key genes. (**A**) ceRNA network. (**B**) TF–mRNA network.

Based on the 35 miRNAs predicted using the miRmap database, 48 lncRNAs were obtained to establish an mRNA–miRNA–lncRNA network, which contained 85 nodes and 197 edges (Fig. **7A**). A total of 270 TFs were predicted using the ChEA3 database, and a TF–mRNA network of the top 30 TFs, *SPHK2*, and *SPNS2* was constructed (Fig. **7B**).

### 3.7 *SPHK2* was Downregulated and *SPNS2* was Upregulated in UC

**Fig. (8):**
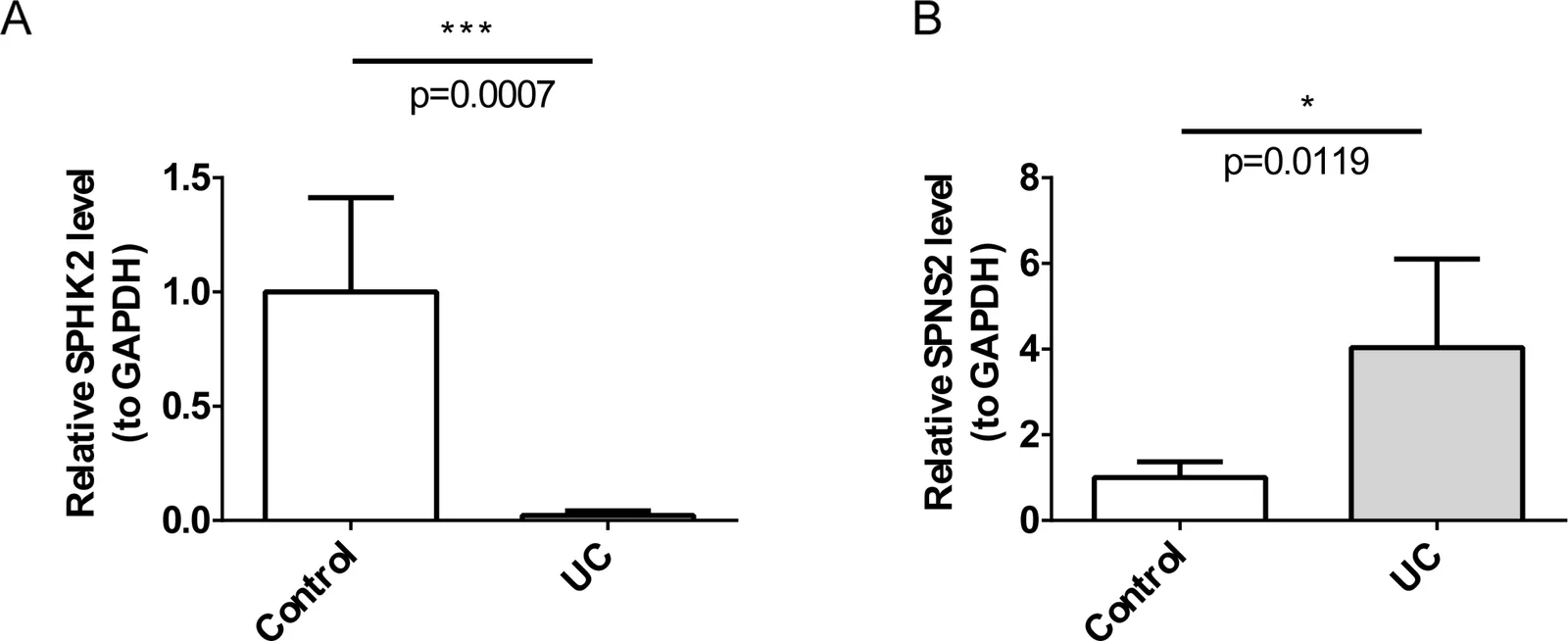
Box plot of the expression of the two key genes evaluated *via* RT-qPCR. (**A**) SPHK2. (**B**) SPNS2. * , *P* < 0.05; ****, *P* < 0.0001.

RT-qPCR showed that *SPHK2* expression was lower and *SPNS2* expression was higher in the UC group. Similar expression trends were observed for both genes in the training and validation sets (Fig. **8**).

## 4 DISCUSSION

Ulcerative colitis (UC), an inflammatory bowel disease, has been extensively studied and is closely associated with cell death [39], lipid metabolism [40], and immune dysregulation [41]. Sphingosine-1-phosphate (S1P) has garnered significant attention in UC research due to its critical role in processes such as cell proliferation, migration, differentiation, and apoptosis [42]. For example, studies by Bram, Verstockt, and others highlight that immune modulation in inflammatory bowel disease (IBD), including UC, is primarily mediated by the S1P signaling pathway and its receptor (S1PR) [43]. Furthermore, S1P receptor modulators such as Ozanimod, Etrasimod, and Tamuzimod have shown efficacy in treating UC [44-47]. These findings emphasize the pivotal role of S1P in UC pathogenesis. However, clinically available S1P-modulating drugs remain limited [45], with existing medications exhibiting certain adverse effects [48] and usage restrictions [45]. Research on S1P in UC is still in its early stages, underscoring the need for further studies with significant clinical implications.

Previous studies on S1P regulation in UC have primarily focused on the S1P receptor S1PR1 [49, 50]. In this study, differential gene expression analysis between UC and control groups revealed significant impacts on immune cell migration, redox regulation, and lipid metabolism pathways. Notably, six genes within the S1P pathway (PNS2, SPHK1, PIK3CG, S1PR1, SPHK2, and S1PR3) exhibited significant differential expression between the two groups. LASSO and SVM-RFE analyses identified SPHK2 and SPNS2 as key genes.

SPHK2 is expressed in various subcellular structures and plays a critical role in regulating several physiological and pathological processes, including cell proliferation [51], migration [52], and inflammation [53]. Studies by Jie, Liang, and Masayuki have demonstrated that SPHK2 deficiency exacerbates chronic intestinal inflammation and colitis-related cancer. This observation aligns with our predictive model, which suggests that lower SPHK2 expression is associated with an increased risk of UC [54]. Moreover, immune infiltration analysis revealed a significant positive correlation between SPHK2 expression and M2 macrophage abundance, underscoring their protective role in UC. In contrast to pro-inflammatory M1 macrophages, M2 macrophages are involved in anti-inflammatory responses and tissue repair. These cells secrete anti-inflammatory cytokines (*e.g.*, IL-10, TGF-β), inhibit antigen presentation, and suppress pro-inflammatory cytokine production (*e.g.*, TNF-α, IL-6) [55, 56]. M2 macrophages also promote extracellular matrix formation, wound healing, and angiogenesis through the secretion of growth factors (*e.g.*, VEGF, FGF, EGF) [57-60]. SPHK2 regulates M2 macrophage differentiation through its enzymatic activity on HDAC, influencing histone acetylation levels [61]. Research by Juan Ji and Juan Wang suggests that SPHK2 facilitates the interaction between KLF4 and HDAC1, induces KLF4 deacetylation, and promotes the transition from M1 to M2 macrophages [62]. In our study, SPHK2 expression was positively correlated with M2 macrophage abundance, further emphasizing their protective role in UC. Gene set enrichment analysis (GSEA) also confirmed the importance of SPHK2 in immune regulation in UC.

Our analysis suggests that SPNS2 may be a potential risk factor for UC, consistent with findings by Ariel L and Burgio [63]. SPNS2 encodes a membrane protein in the Spinster family [64] and is involved in signal transduction, lipid transport, and cell communication. As a transporter of S1P, SPNS2 facilitates its movement from intracellular to extracellular spaces [65]. Subcellular localization analysis revealed that SPNS2 is predominantly located in the plasma membrane and endosomes, suggesting its role in transmembrane signal transduction. Additionally, immune infiltration analysis showed a significant positive correlation between SPNS2 expression and neutrophil abundance. GSEA highlighted the role of SPNS2 in cytokine-cytokine receptor interactions, chemotaxis, and metabolic processes. Under hypoxic conditions, SPNS2 enhances NADPH oxidase (phox) activation in phagocytes and reactive oxygen species (ROS) production, contributing to the formation of neutrophil extracellular traps (NETs) [66] and playing a crucial role in sustaining the inflammatory cytokine response in UC by promoting TNF-α and IL-1β production [67]. Although research on SPNS2 in UC remains limited, its central role in S1P signaling and immune regulation positions it as a key target for future UC research.

Furthermore, the transcriptional regulatory network established in this study demonstrates that both SPHK2 and SPNS2 are regulated by a variety of transcription factors (TFs) and microRNAs (miRNAs), suggesting a close association between the gene expressions of SPHK2 and SPNS2. GeneMANIA analysis revealed that these genes interact with various inflammatory cytokines (*e.g.*, IL12RB2, IL12RB1, IL12A), cell proliferation markers (*e.g.*, AGK, BRCA2, MAPKAPK2), and sphingolipid metabolism-related genes (*e.g.*, CERK, PLPP1, SGPP1). Therefore, SPHK2 and SPNS2 appear to have biological functions extending beyond immune regulation, underscoring the need for further investigation into their transcriptional regulation and downstream mechanisms. This study identified two potential therapeutic targets through the analysis of publicly available datasets. However, several limitations should be acknowledged. First, given the reliance on public data and the relatively small sample size, potential biases in data completeness and quality cannot be excluded. The functional roles of these genes are currently inferred solely based on computational analyses, and their biological functions in vivo require further experimental validation. Second, this study focused exclusively on mRNA expression levels and did not investigate potential post-translational modifications, which may significantly influence protein function and activity. Future studies should consider these modifications and employ experimental approaches, such as animal model construction and Western blotting, to elucidate the underlying molecular mechanisms. While this research provides a preliminary theoretical foundation for target identification, further comprehensive and rigorous investigations are necessary to address the aforementioned limitations and facilitate clinical translation.

## CONCLUSION

This study, based on data from different cohorts in the GEO database, employed methods such as differential gene enrichment and machine learning to identify SPHK2 and SPNS2 as key genes in UC. SPHK2, acting as a protective factor, is lowly expressed in UC patients, and its downregulation may suppress the abundance of M2 macrophages, while SPNS2, acting as a risk factor, is highly expressed and may induce the formation of NETs. The combined effect of these two genes may lead to increased intestinal inflammation in UC patients.

## Data Availability

The data and supportive information are available within the article.

## LIST OF ABBREVIATIONS

UC: Ulcerative Colitis
DEGs: Differentially Expressed Genes
S1P: Sphingosine-1-phosphate
SPHK1: Sphingosine kinase 1
SPHK2: Sphingosine-kinase 2
PPI: Protein–Protein Interaction
ROC: Receiver operating characteristic
AUC: Under the Curve
TFs: Transcription Factors
TNF: tumor necrosis factor
IBD: Inflammatory Bowel Disease
